# Genome-wide nucleosome mapping in multiple myeloma

**DOI:** 10.1186/1756-8935-6-S1-P124

**Published:** 2013-04-08

**Authors:** Eliza C Small, Quanwei Zhang, Relja Popovic, Youjia Hua, Ji-Ping Wang, Jonathan D Licht

**Affiliations:** 1 Division of Hematology/Oncology, Northwestern University Feinberg School of Medicine, Chicago, IL, USA; 2 Department of Statistics, Northwestern University, Evanston, IL, USA

## 

Proper regulation of gene expression is essential for cellular viability, and the integrity of chromatin is a fundamental factor in maintaining proper gene regulation. In cancer cells, this precise regulation is disrupted and aberrant gene expression ensues - a hallmark of the disease. In many forms of hematological malignancy, aberrant expression, activation or deletion of a critical enzymatic regulator of chromatin modification underlies the disease. One such regulator is MMSET (multiple myeloma SET domain) protein, a histone methyltransferase, that is rearranged and joined with the immunoglobulin locus associated with t(4:14) due to aberrant class switch DNA recombination in approximately 20% of multiple myeloma cases and is associated with particularly poor prognosis. Our lab has previously shown that an increase in MMSET expression results in global histone modification changes and up-regulation of hundreds of genes. We believe that these changes are due to radical reprogramming of chromatin, including repositioning of nucleosomes in MMSET-overexpressed cells. Mechanistic insight into epigenetic changes during MMSET-mediated gene expression will provide deeper understanding of disease progression in multiple myeloma, and potentially other similar malignancies.

To begin understanding the changes in chromatin structure when MMSET is overexpressed, we generated a genome wide nucleosome map in cells expressing high and low levels of MMSET. Initial analysis of this mapping reveals changes in nucleosome positioning in promoters where gene expression and histone modifications had been identified to be significantly altered in cells expressing high and low levels of MMSET. Generally, overexpression of MMSET results in an increase in H3K36 dimethylation, a histone modification associated with actively transcribed genes, and a concomitant genome-wide loss of H3K27 trimethylation, a histone modification associated with repressed genes. Indeed, in cells expressing high levels of MMSET, genes that have been identified to have an increase in expression and an increase in H3K36 dimethylation have a distinct change in nucleosome positioning compared to the cells expressing low levels of MMSET. Further, differences in nucleosome positioning can be detected in promoter regions with increased H3K27 trimethylation and decreased gene expression. We are currently examining whether these changes in nucleosome positioning are due to the presence of MMSET and require MMSET histone methyltransferase activity. Through these and related studies, our aim is to determine how MMSET behaves in multiple myeloma through a likely radical reprogramming of chromatin. We believe that mechanistic insight into MMSET-mediated gene expression changes will provide significant avenues for potential therapeutic strategies, as MMSET overexpression has been implicated in myeloma and other solid tumors. Moreover, our work will provide insight into mechanisms that may be shared amongst various diseases that are initiated through epigenetic misregulation.

